# Common scab disease: structural basis of elicitor recognition in pathogenic *Streptomyces* species

**DOI:** 10.1128/spectrum.01975-23

**Published:** 2023-10-04

**Authors:** Frédéric Kerff, Samuel Jourdan, Isolde M. Francis, Benoit Deflandre, Silvia Ribeiro Monteiro, Nudzejma Stulanovic, Rosemary Loria, Sébastien Rigali

**Affiliations:** 1 InBioS–Center for Protein Engineering, Institut de Chimie B6a, University of Liège, Liège, Belgium; 2 Department of Biology, California State University, Bakersfield, California, USA; 3 Department of Plant Pathology, University of Florida, Gainesville, Florida, USA; University of Minnesota Twin Cities, St. Paul, Minnesota, USA

**Keywords:** host-pathogen interaction, plant pathogens, ligand-protein interaction, sugar transport, carbohydrate metabolism, elicitor binding, *Streptomyces*

## Abstract

In *Streptomyces scabiei*, the main causative agent of common scab disease of root and tuber crops, the interaction between the substrate-binding protein (SBP) CebE (CebE^scab^) and cellotriose released by the plant host (*K*_*D*_ in the nanomolar range) is the first event for the onset of its pathogenic lifestyle. Here, we report the structure of CebE^scab^ in complex with cellotriose at a resolution of 1.55 Å, adopting a general fold of the B subcluster of SBPs. The interaction between CebE^scab^ and cellotriose involves multiple direct or water-mediated hydrogen bonds and hydrophobic interactions, with the glucose monomer at the non-reducing end occupying the most conserved part of the substrate-binding cleft. As main interactions between the two domains of CebE involve cellotriose itself, the closed conformational state of CebE is performed via an induced-fit ligand binding mechanism where cellotriose binding triggers the domain movement. Analysis of regulon predictions revealed that the signaling pathway from CebE-mediated cellotriose transport to the transcriptional activation of thaxtomin phytotoxin biosynthesis is conserved in *Streptomyces* spp. causing common scab, except for *Streptomyces ipomoeae,* which specifically colonizes sweet potatoes and responds to other and yet unknown virulence elicitors. Interestingly, strains belonging to the pathogenic species *turgidiscabies* and *caniscabiei* have a cellotriose-binding protein orthologous to the CebE protein of the saprophytic species *Streptomyces reticuli* with lower affinity for its substrate (*K*_*D*_ in the micromolar range), suggesting higher cellotriose concentrations for perception of their host. Our work also provides the structural basis for the uptake of cellobiose and cellotriose by non-pathogenic cellulose-decomposing *Streptomyces* species.

**IMPORTANCE:**

Common scab is a disease caused by a few *Streptomyces* species that affects important root and tuber crops including potato, beet, radish, and parsnip, resulting in major economic losses worldwide. In this work, we unveiled the molecular basis of host recognition by these pathogens by solving the structure of the sugar-binding protein CebE of *Streptomyces scabiei* in complex with cellotriose, the main elicitor of the pathogenic lifestyle of these bacteria. We further revealed that the signaling pathway from CebE-mediated transport of cellotriose is conserved in all pathogenic species except *Streptomyces ipomoeae,* which causes soft rot disease in sweet potatoes. Our work also provides the structural basis of the uptake of cellobiose and cellotriose in saprophytic *Streptomyces* species, the first step activating the expression of the enzymatic system degrading the most abundant polysaccharide on earth, cellulose.

## INTRODUCTION

Microbes interacting with plants, either beneficial or pathogenic, must perceive molecules indicating the presence of their host to trigger the appropriate response for tissue penetration and colonization (1). Protein-ligand interactions are also often the starting event for saprophytic microorganisms, which must sense byproducts released from the decaying plant material to switch on the expression of specific carbon source uptake and catabolic systems. For organic soil-dwelling bacteria, lignocellulose is a major nutrient reservoir, first by itself for being the most abundant polysaccharide on earth, but also because crystalline cellulose is physically linked to other important nourishing polymers such as xylan, mannan, pectin, and lignin. Most members of the bacterial genus *Streptomyces* have acquired a complete cellulolytic system that comprises structurally diverse and synergistically acting secreted cellulose-degrading enzymes to generate, import, and consume cellobiose and other cello-oligosaccharides (2 – 5). In these species, cellobiose—the main carbohydrate released by the cellulolytic system (6 – 8)—and cellotriose, are actively imported by the CebEFG-MsiK ATP-binding cassette (ABC) transporter (9 – 12). CebE is the sugar-binding component of the ABC transporter, proteins CebF and CebG form the transporter permease, and the energy for active cello-oligosaccharide transport is provided by the multiple sugar importer ATPase MsiK. The imported cello-oligosaccharides are subsequently hydrolyzed by the main beta-glucosidase BglC, and/or the alternative beta-glucosidase BcpE1 (13) to feed the glycolysis directly with glucose (14). The use of carbohydrates emanating from cellulose degradation appears to be so crucial for catabolism that multiple copies of the *cebR-cebEFG-bglC* gene cluster are often present in the genomes of these organisms, either acquired by horizontal transfer (xenologs) or by gene duplication (paralogs) (3, 10, 13, 15 – 17).

For *Streptomyces scabiei* (syn. *scabies*) and other *Streptomyces* species producing the thaxtomin phytotoxins responsible for the disease called common scab on root and tuber crops, cello-oligosaccharides emanating from the plant cell wall are not only perceived as nutrients but also as signals for triggering their pathogenic lifestyle (9, 15, 18 – 20). Indeed, the production of thaxtomins and other key metabolites of the virulome is activated by the transport of cello-oligosaccharides, particularly cellotriose (15, 18, 21, 22). The binding-affinity of CebE of *S. scabiei* (CebE^scab^) for cello-oligosaccharides has *K*_*D*_ values of 14 (±2) nM and 2 (±0.5) nM for cellobiose and cellotriose, respectively (9). Instead, the affinity for cello-oligosaccharides of the CebE protein of the highly cellulolytic species *Streptomyces reticuli* (CebE^reti^) is much lower with *K*_*D*_ at the micromolar level (23). The high affinity of CebE^scab^ to cello-oligosaccharides would make *S. scabiei* one of the first beneficiaries of the cello-oligosaccharides released by efficient lignocellulolytic microorganisms. Yet, for this strain that evolved to a disabled cellulolytic system (18, 24), it would be crucial to be able to distinguish between cello-oligosaccharides from living and decaying plant material. We postulated that the particularly high affinity of CebE^scab^ for cellotriose could be a key feature of *S. scabiei* for discerning living plants from plant decaying material and, therefore, to adopt either a pathogenic or saprophytic lifestyle (24). Indeed, cellobiose is by far the main product released by the cellulolytic systems (6 – 8), while Johnson et al. detected only cellotriose released from rapidly growing radish seedlings and from actively dividing tobacco NT1 cells in suspension (18). Therefore, cellotriose is thought to be perceived as the signal molecule specifying *S. scabiei* the nearby presence of a growing host to colonize, whereas sensing cellobiose would indicate *S. scabiei* dead plant material to consume (15, 24). In addition, *S. scabiei* species possess the alternative CebEFG2 ABC-transporter system that also participates in the uptake of cello-oligosaccharide elicitors (17).

In this work, we elucidated the structure of CebE^scab^ in complex with cellotriose thereby identifying the key residues involved in elicitor recognition for the onset of the pathogenic lifestyle of *S. scabiei* and other phytopathogenic *Streptomyces* species.

## MATERIALS AND METHODS

### Strains, chemicals, and culture conditions

*Escherichia coli* DH5α was used for routine molecular biology applications, and *Escherichia coli* BL21(DE3) Rosetta (Novagen) for heterologous protein production. *E. coli* strains were cultured in lysogeny broth (LB) (BD Difco LB broth) medium supplemented with the appropriate antibiotics [kanamycin (50  µg/mL), chloramphenicol (25  µg/mL)]. Cellotriose was purchased from Carbosynth.

### Production and purification of CebE from *S*. *scabiei* 87-22

Heterologous production of CebE^scab^ (SCAB_57751; WP_013003368) was performed in strain *E. coli* BL21(DE3) Rosetta (Novagen) harboring pSAJ016 [pET28a derivative containing the coding sequence of *scab57751* (*cebE*) without the first 132 nt—corresponding to the signal peptide—inserted into NdeI and HindIII restriction sites (9)]. Production and purification by nickel affinity chromatography were performed as previously described (9).

### Crystallization and structure determination of CebE^scab^ in complex with cellotriose

CebE^scab^ was concentrated to 15 mg/mL in a Tris-HCl 30 mM pH 7.5 buffer containing 150 mM NaCl. Cellotriose was added to a 15-mM final concentration. Crystals were obtained using the sitting-drop vapor diffusion method at 4°C with drops made of 0.2 µL of protein solution mixed with 0.2 µL of precipitant solution [polyethylene glycol 3350 25% (wt/vol), and sodium citrate buffer 0.1 M pH 3.5]. The crystal was transferred into a cryoprotectant solution containing 45% (vol/vol) glycerol and 20% (wt/vol) polyethylene glycol 6000 before flash-freezing in a liquid nitrogen bath. Diffraction data were collected at the Soleil Synchrotron PROXIMA 2A beamline (Paris) using a Dectris Pilatus 6M detector. The wavelength and temperature of data collection were 0.9786 Å and 100 K, respectively. The first 100° (500 frames of 0.2°) of two data sets were integrated and scaled together using XDS (25). The data were deposited in the SBGrid Data Bank (https://doi.org/10.15785/SBGRID/1035). Initial phases were obtained by molecular replacement with the AlphaFold (26) model of CebE^scab^ as a search model using Phaser (27). The structure was built with Coot (28) and refined with Refmac (29). The figures were prepared using PyMOL (The PyMOL Molecular Graphics System, Version 2.4.1 Enhanced for Mac OS X, Schrödinger, LLC.). The CebE structure in complex with cellotriose can be found at PDB DOI:https://www.rcsb.org/structure/8BFY.

### Regulon predictions

Computational prediction of CebR binding sites was performed with the PREDetector software (30) according to the methodology and philosophy described in reference (31). Sequences used to generate the CebR position weight matrix are listed in Table S1.

## RESULTS AND DISCUSSION

### Overall three-dimensional structure of CebE^scab^ binding cellotriose

We obtained the crystallographic structure of CebE^scab^ in complex with cellotriose at a 1.55-Å resolution. The crystal belongs to the P2_1_ space group with one molecule in the asymmetric unit. The final *R*_work_ and *R*_free_ are 13.6% and 17.8%, respectively (Table 1). The CebE^scab^ structure contains residues 62–454. A single segment of 33 amino acids at the N-terminus, which includes 17 residues from the CebE sequence and 16 residues from the His-Tag used for purification, has not been modeled because of the lack of electron density.

**TABLE 1:** Data collection and refinement statistics

	CebE^scab^-cellotriose
PDB code	8BFY
Data collection	
Wavelength	0.9786
Space group	P 2_1_
a, b, c (Å)	59.39, 39.43, 79.64
α, β, γ (°)	90, 92.38, 90
Resolution range (Å)^*a*^	35.3–1.55 (1.59–1.55)
*R*_merge_ (%)^*a*^	7.3 (111.9)
*R*_meas_ (%)^*a*^	8.4 (133.3)
<I> /<σI> ^*a*^	13.1 (1.3)
Completeness (%)^*a*^	99.2 (97.1)
Redundancy ^*a*^	3.8 (3.1)
CC 1/2^*a*^	0.999 (0.524)
Refinement	
No. of unique reflections	53,542
*R*_work_ (%)	13.6
*R*_free_ (%)	17.8
No. of non-H atoms	
Protein	3,019
Ligand	34
Solvent	374
RMS deviations from ideal stereochemistry	
Bond lengths (Å)	0.007
Bond angles (^o^)	1.37
Mean B factor (Å^2^)	
Protein	23.7
Ligand	16.0
Solvent	37.1
Ramachandran plot	
Favored region (%)	98.7
Allowed regions (%)	1.3
Outlier regions (%)	0.0

^*a*^ Numbers in parenthesis refer to the highest resolution shell.

**Fig 1:**
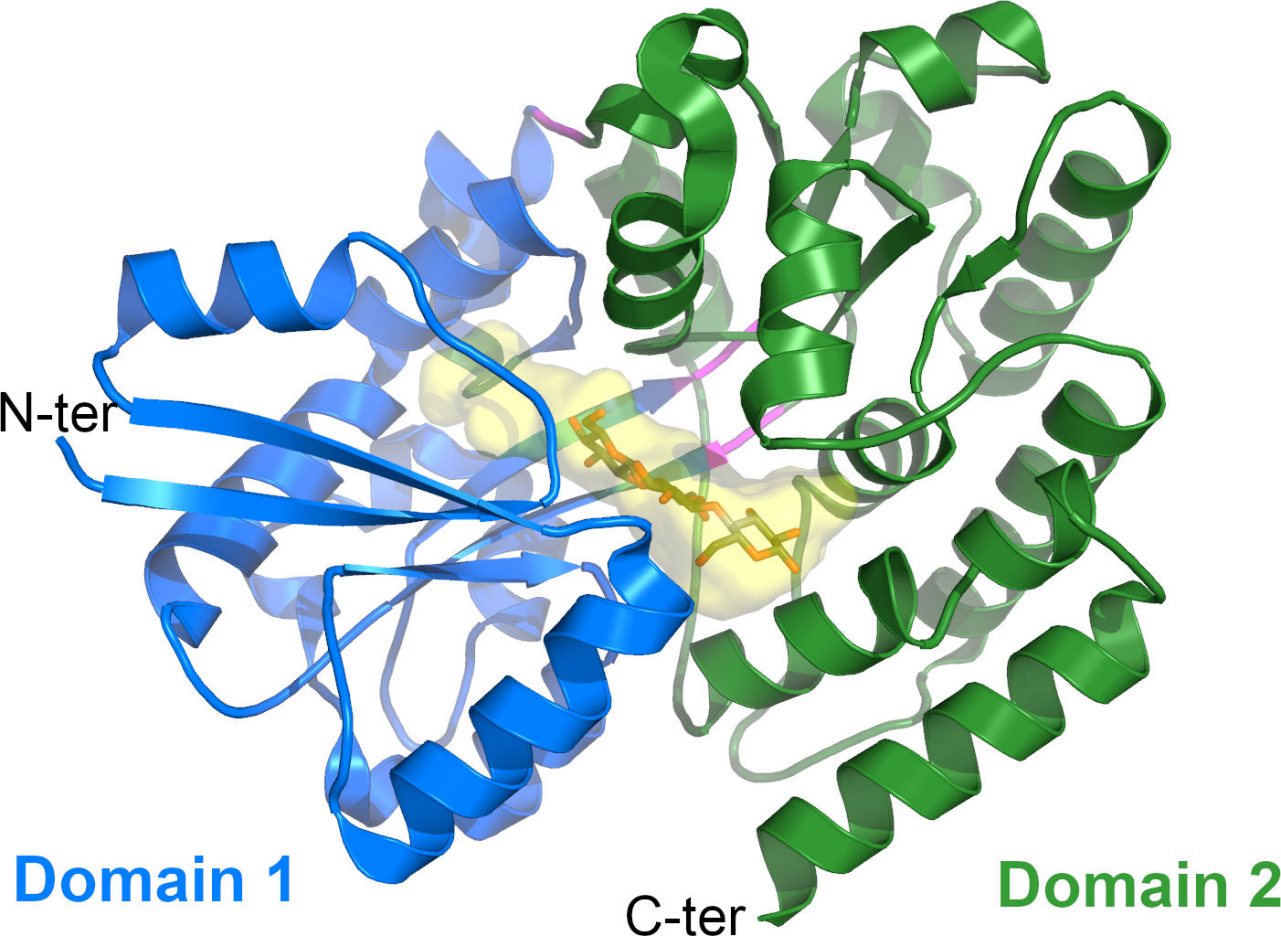
Overall structure of the CebE/cellotriose complex. Cartoon representation of CebEscab with Domains 1 and 2 in blue and green, respectively. The three hinge regions are colored in magenta. The closed cavity occupied by cellotriose (gray sticks) is shown as a yellow transparent surface.

CebE^scab^ adopts a cluster B-type substrate-binding protein (SBP)-fold as described by Berntsson et al. (32, 33). It is composed of two domains connected by three hinge regions. Domain 1 contains residues 62–176 and 330–386 and is made of a six-stranded β-sheet surrounded by 10 helices (Fig. 1). The larger Domain 2 includes residues 177–329 and 387–454 and is formed by a four-stranded β-sheet surrounded by 10 helices with the two C-terminal helices packed on it. The elongated ligand binding pocket is located at the interface between the two domains (Fig. 1).

### The cellotriose binding site of CebE^scab^

**Fig 2:**
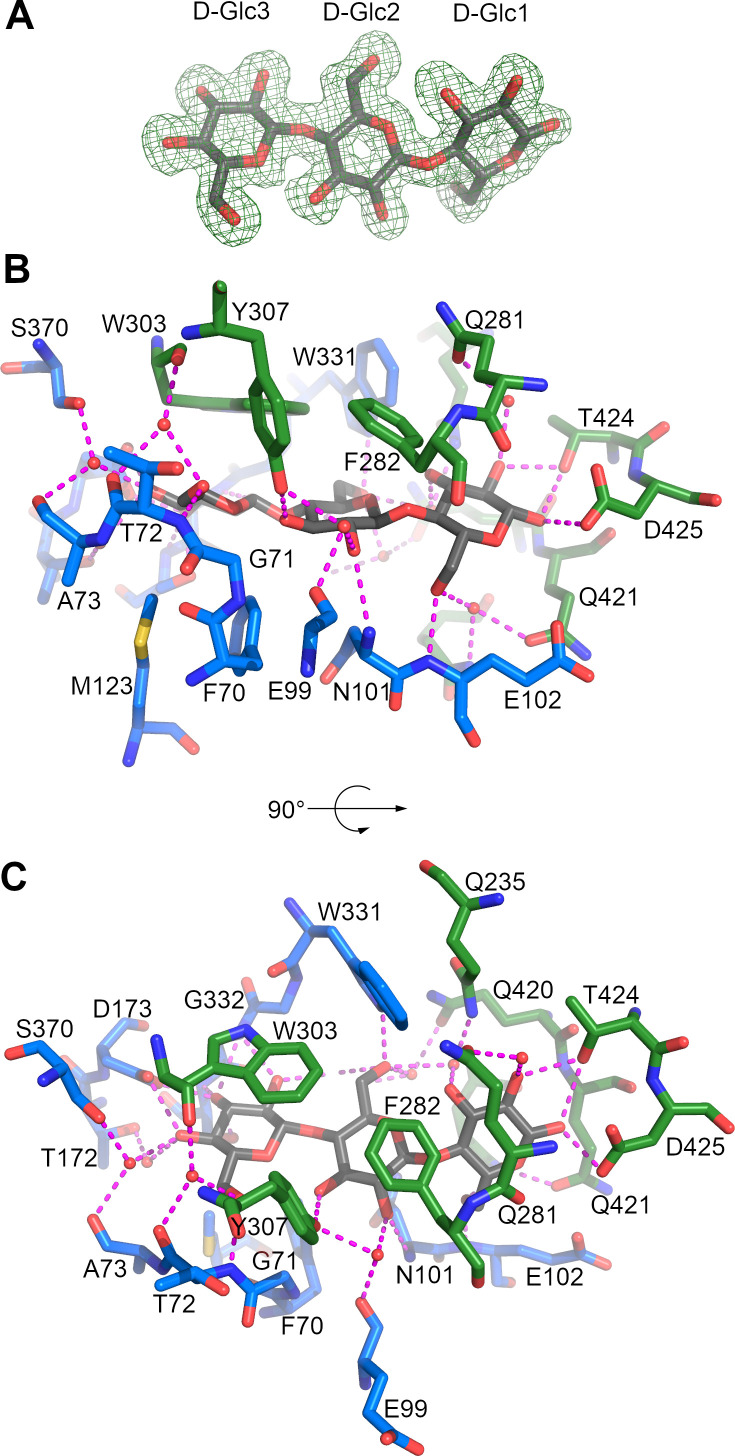
Substrate binding site of the CebE:cellotriose complex. (A) OMIT electron density map displayed at 1 σ level around cellotriose. (B) Interactions stabilizing cellotriose (gray sticks), residues from Domains 1 and 2 are displayed as blue and green sticks, respectively, water molecules as small red spheres, and H-bonds as magenta dashed lines. (**C**) Same as panel (B) with a 90° rotation around a horizontal axis.

CebE^scab^ was crystallized in the presence of 15 mM cellotriose, which approximately corresponds to a 50-fold excess compared to the protein concentration. An electron density corresponding to the whole cellotriose molecule is observed in the ligand binding pocket (Fig. 2A), clearly establishing the two β-1,4 links between the three β-D-glucoses. The reducing end (D-Glc1) is more inserted in Domain 2, whereas the non-reducing end (D-Glc3) makes more interactions with Domain 1 (Table 2). Cellotriose is stabilized in the pocket by a few hydrophobic interactions and numerous H-bonds, nine of them being mediated by eight water molecules surrounding the ligand (Fig. 2B and C; Table 2). D-Glc3 provides the highest contribution to the binding of cellotriose, being involved in ten H-bonds and three hydrophobic interactions with the sidechain of W303 (parallel stacking), F70, and M123. D-Glc1 and D-Glc2 follow with 7 and 6 H-bonds, respectively, the latter being involved in an additional hydrophobic interaction with F282.

**TABLE 2:** List of interactions between CebE^scab^ and cellotriose

Cellotriose monomer	Cellotriose atom	CebE domain	CebE residue	CebE atom	Distance (Å)	Type of interaction
D-Glc1	O1	2	D425	OD2	2.8	H-bond
	O1	2	T424	OG1	3.1	H-bond
	O2	2	T424	OG1	2.8	H-bond
	O2	2	Q281	OE1	2.8/2.7	H_2_O-mediated H-bond^*a*^
	O3	2	Q235^*b*^	NE2	2.8/3.0	H_2_O-mediated H-bond^*a*^
	O6	1	E102^*b*^	N	2.8	H-bond
	O6	2	Q417^*b*^ (Q421)	NE2 (OE1)	2.7/2.9 (2.6)	H_2_O-mediated H-bond^*a*^
D-Glc2	O2	1	N101^*b*^	N	3.2	H-bond
	O2	1	E99^*b*^ (Y307)	O (OH)	2.7/2.7 (2.9)	H_2_O-mediated H-bond^*a*^
	O3	2	Y307^*b*^	OH	2.7	H-bond
	O6	1	W331	NE2	2.9	H-bond
	O6	2	Q420^*b*^ (E125)	OE1 (OE1)	3.1/2.7 (2.6)	H_2_O-mediated H-bond^*a*^
	O6	2	Q235^*b*^	NE2	2.9/3.0	H_2_O-mediated H-bond^*a*^
	Ring	2	F282	Phenyl	4.7	Hydrophobic
D-Glc3	O2	1	E125	OE2	2.7	H-bond
	O2	1	G333	N	2.9	H-bond
	O3	1	G333	N	3.0	H-bond
	O3	1	D173	OD1	2.6	H-bond
	O3	1	S334	OG	2.7	H-bond
	O4	1	D173	OD2	2.7	H-bond
	O4	1	T172	OG1	3.0/2.8	H_2_O-mediated H-bond^*a*^
	O4	1	S370^*b*^ (A73)	OG (O)	2.8/2.5 (2.9)	H_2_O-mediated H-bond^*a*^
	O6	1	T72^*b*^	N	2.8	H-bond
	O6	2	W303^*b*^ (T72)	O (O)	2.7/2.7 (2.9)	H_2_O-mediated H-bond^*a*^
	Ring	2	W303	Indole	4.1	Hydrophobic
	Ring	1	F70	Phenyl	3.7	Hydrophobic
	Ring	1	M123^*b*^	CE	4.8	Hydrophobic

^*a*^ When H_2_O mediates H-bond, the first distance is related to the cellotriose-H_2_O bond and the second to the H_2_O-protein bond. Parentheses are used when a second residue of the protein contributes to the binding of the H_2_O molecule.

^*b*^ The residues of CebE^scab^ that are substituted in CebE^reti^.

The importance of D-Glc3 for the affinity of cellotriose is further highlighted by comparing the CebE^scab^:cellotriose structure with the closest structure of SBP proteins in complex with a ligand available in the Protein Data Bank (Fig. S1): the ABC transporter-associated binding protein from *Bifidobacterium animalis* (Bal6GBP) in complex with β-1,6-galactobiose [PDB code 6H0H, 26% of sequence identity (34)], the ABC transporter-associated binding protein AbnE from *Geobacillus stearothermophilus* in complex with arabinohexaose [PDB code 6RKH, 26% of sequence identity (35)], and the galacto-N-biose-/lacto-N-biose I-binding protein of the ABC transporter from *Bifidobacterium longum* in complex with lacto-N-tetraose (36). Indeed, despite the difference in length and composition of the different oligosaccharides present in the three structures, a saccharide is always bound at a position equivalent to that of D-Glc3, and two residues important for its binding, D173 and W303, are conserved in the structure of these four different ABC-type sugar-binding proteins (Fig. S1), as well as in CebE2, the alternative cello-oligosaccharide transporter of *S. scabiei* strains (17). While there seems to be a preference for the nonreducing end of the oligosaccharide, it is not exclusive as illustrated by the AbnE:arabinohexaose complex in which it is the fifth arabinose that is bound at this conserved binding position.

### Substrate-induced closing of the CebE^scab^ pocket

In the CebE^scab^:cellotriose structure, only seven H-bonds are observed between residues of Domains 1 and 2 when the three hinge regions are removed (P176-M177, G329-N330, and A386-K387), involving residues R100, G127, N128, E131, W331, and Q368 for Domain 1, and Q281, F282, W303, K309, K415, and Q417 for Domain 2. In addition, the two significant hydrophobic interactions between the domains are located in the vicinity of the three hinge regions (P302, F400, and I396 with F365 and F371 for the first hydrophobic cluster, and V409 and I407 with W155 for the second). It, therefore, seems that an important part of the interactions between Domains 1 and 2 is mediated by the cellotriose molecule itself. The ligand would, therefore, be responsible for the closing of the sugar-binding pocket, a phenomenon termed as induced-fit ligand binding mechanism (37). This is compatible with the known flexibility of these domains around the hinge regions (35), which is necessary for the cavity to open and allow access to the ligand. Indeed, in the CebE^scab^:cellotriose structure, the pocket accommodating the cellotriose molecule has no access to the solvent (Fig. 1B).

**Fig 3:**
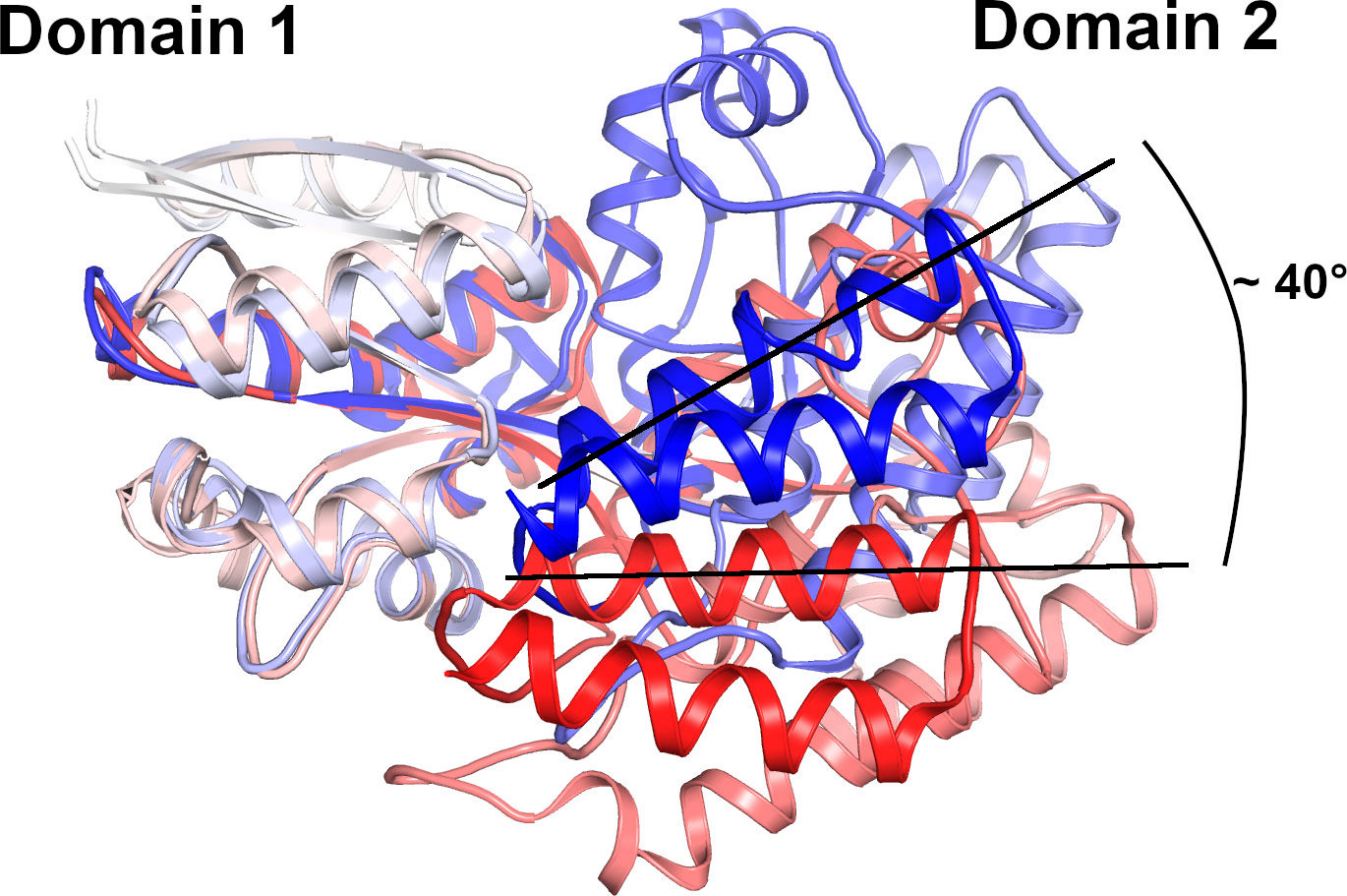
Conformational change of CebE upon cellotriose binding. Superimposition of the CebEscab:cellotriose structure (colored with a gradient from the white N-terminus to the blue C-terminus) to the KfSBP structure in an open conformation (colored with a gradient from the white N-terminus to the red C-terminus); only Domain 1 residues were used to calculate the superimposition. Note the ±40° angle corresponding to the movement of Domain 2 upon cellotriose binding.

A good estimate of the magnitude of the opening can be obtained by comparing our closed CebE^scab^ structure with the structure of the solute binding domain protein from *Kribbella flavida* DSM 17836 (KfSBP, PDB code 5IXP) (Fig. 3). KfSBP is the closest homolog of CebE^scab^ in the Protein Data Bank and shares 38% of sequence identity with CebE^scab^, including most of the interactions with cellotriose. Nine residues with sidechain interacting with the ligand differ between KfSBP and CebE^scab^: the M123A mutation is compensated by the A73F one, the F282W substitution provides a more extended hydrophobic interaction, but it is conjugated with the Y307V mutation that induces the loss of a H-bond with cellotriose, the D425E difference should maintain the H-bond, and the Q235E, Q281A, Q417G, and Q420N substitutions induce modifications of the water-mediated H-bond network, which are difficult to quantify. When the two Domain 1 of the two proteins are superimposed, the two Domain 2 are separated by a rotation around the hinge regions of approximately 40° (Fig. 3), inducing a large opening of the ligand binding pocket. The extent of the conformational change is better perceived with Movie S1 displaying a morphing between the CebE^scab^:cellobiose structure and a CebE^scab^ model obtained using the comparative modeling method RosettaCM (38) with the KfSBP structure as a template. The magnitude of the conformational change of CebE^scab^ upon ligand binding could be further validated by small-angle X-ray scattering experiments as previously done with AbnE (35).

### Conservation of the cellotriose-mediated signaling pathway in pathogenic *Streptomyces* species

**Fig 4:**
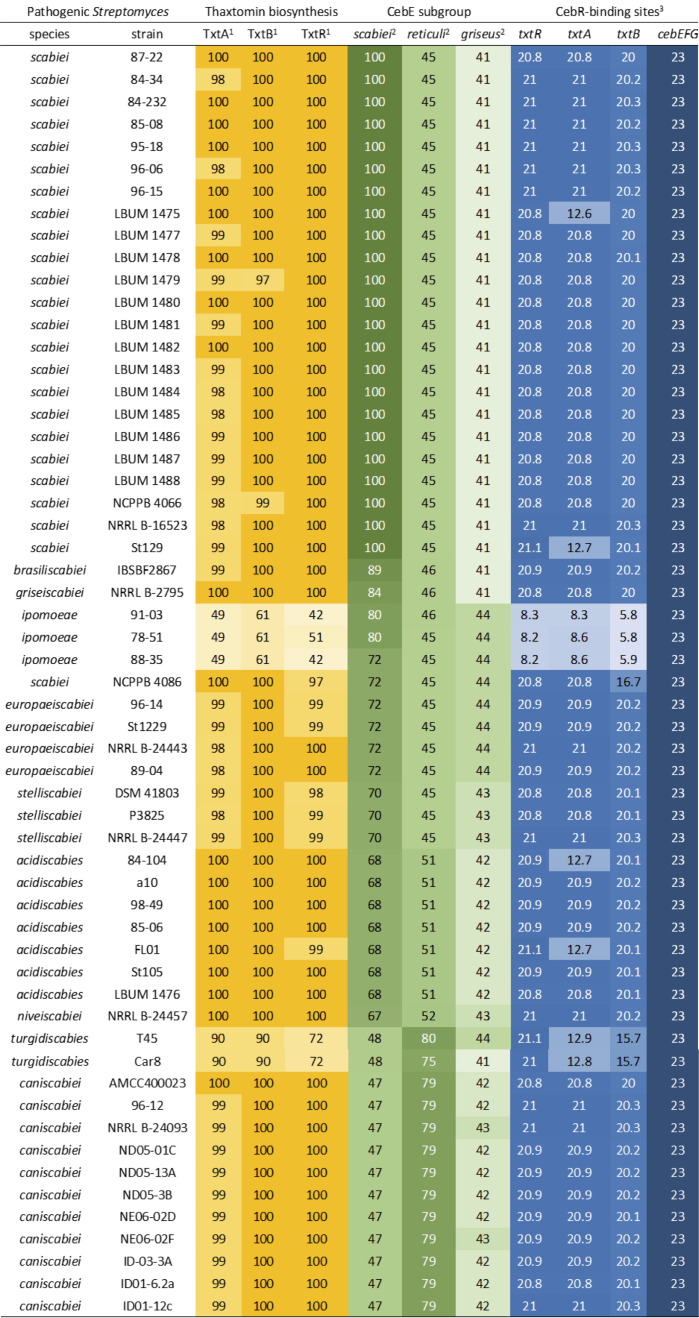
Type of cellotriose/CebE-mediated signaling pathways to thaxtomin production. 1, the values refer to the amino acid identity expressed in percentage compared to the proteins of *S. scabiei* 87–22 used as reference sequences. 2, the values refer to the amino acid identity expressed in percentage compared to the CebE proteins of *S. scabiei* 87–22 (WP_013003368.1), *S. reticuli* (CAB46342.1), and *S. griseus* (WP_012379731.1). 3, the values refer to the score obtained for a 14-nt sequence according to the position weight matrix generated from the experimentally validated CebR-binding sites (23 is the maximum score corresponding to the 14-nt TGGGACGCGTCCCA palindromic sequence).

The ABC transport systems for cello-oligosaccharide import by both pathogenic and saprophytic *Streptomyces* species are clustered in at least four different paralog/xenolog subgroups (17), namely CebE^scab^ (9), CebE2^scab^ (17), CebE^reti^ (10, 23), and CebE^gris^ (16). CebE^scab^ and CebE^reti^ are able to bind cellotriose at nano- and micro-molar ranges, respectively, while the *K*_*D*_ values of CebE^gris^ and CebE2^scab^ for cellobiose and cello-oligosaccharides have not been experimentally determined (9, 10). The genes required for producing thaxtomin phytotoxins, which are included in the pathogenicity island that has been horizontally transferred to different saprophytic *Streptomyces* species, have therefore been integrated into genomes with different backgrounds regarding the affinity of the CebE protein for its substrates. Figure 4 lists all thaxtomin-producing pathogenic *Streptomyces* species (57 strains from 10 different species) for which a good quality genome sequence was available and therefore where we could identify the type(s) of CebE protein(s) involved in cellotriose and cellobiose uptake. In addition, the assessment of the conservation of the cellotriose-mediated induction of pathogenicity in all selected strains was performed by screening for binding sites of the transcriptional repressor for cellulose and cello-oligosaccharide utilization CebR in the biosynthetic gene cluster associated with thaxtomin production (*txt* cluster), and within the *cebEFG* operon. Two main groups can be distinguished, i.e., the species that possess CebE^scab^ (44 strains from 8 different species), and those that possess CebE^reti^ (13 strains from 2 species). Surprisingly, none of the pathogenic strains (with genome available) recruited the CebE-like protein of *Streptomyces griseus* group (16) as elicitor importer. The CebE^scab^ group includes strains that belong to species *S. scabiei*, *Streptomyces acidiscabies*, *Streptomyces europeiscabiei*, *Streptomyces stelliscabiei*, *Streptomyces brasiliscabiei*, *Streptomyces griseiscabiei*, and *Streptomyces niveiscabiei* (Fig. 4). *Streptomyces ipomoeae* is also part of this group, but the absence of a CebR-binding site within the *txt* cluster would explain earlier results that suggested this species did not select the CebE-cello-oligosaccharide-mediated pathway for the induction of thaxtomin production (39, 40). The CebE^reti^ group includes the strains that belong to species *Streptomyces turgidiscabies* and *Streptomyces caniscabiei* (Fig. 4).

Based on *K*_*D*_ values measured for two different CebE protein subgroups, pathogenic *Streptomyces* species would trigger thaxtomin production after sensing cellotriose at either the nano- (CebE^scab^ subgroup) or at the micro-molar level (CebE^reti^ subgroup). This major difference in the CebE affinity for the natural elicitor cellotriose may result in species sensitive to different concentration thresholds for the molecules eliciting their pathogenic lifestyle. The molecular origin of this significant difference of affinity can be explored using the AlphaFold (26) model of CebE^reti^ available in the AlphaFold database (UniProt id Q9 × 9R7_STRRE). This model is of very good quality with an average pLDDT (predicted Local Distance Difference Test) value of 94.3 calculated for the Cα of the globular part of the protein (from I51 to Q444). The CebE^reti^ model corresponds to the closed conformation of the protein and can be very well superimposed to CebE^scab^ (root mean square deviation of 1.4 Å calculated over 387 Ca).

**Fig 5:**
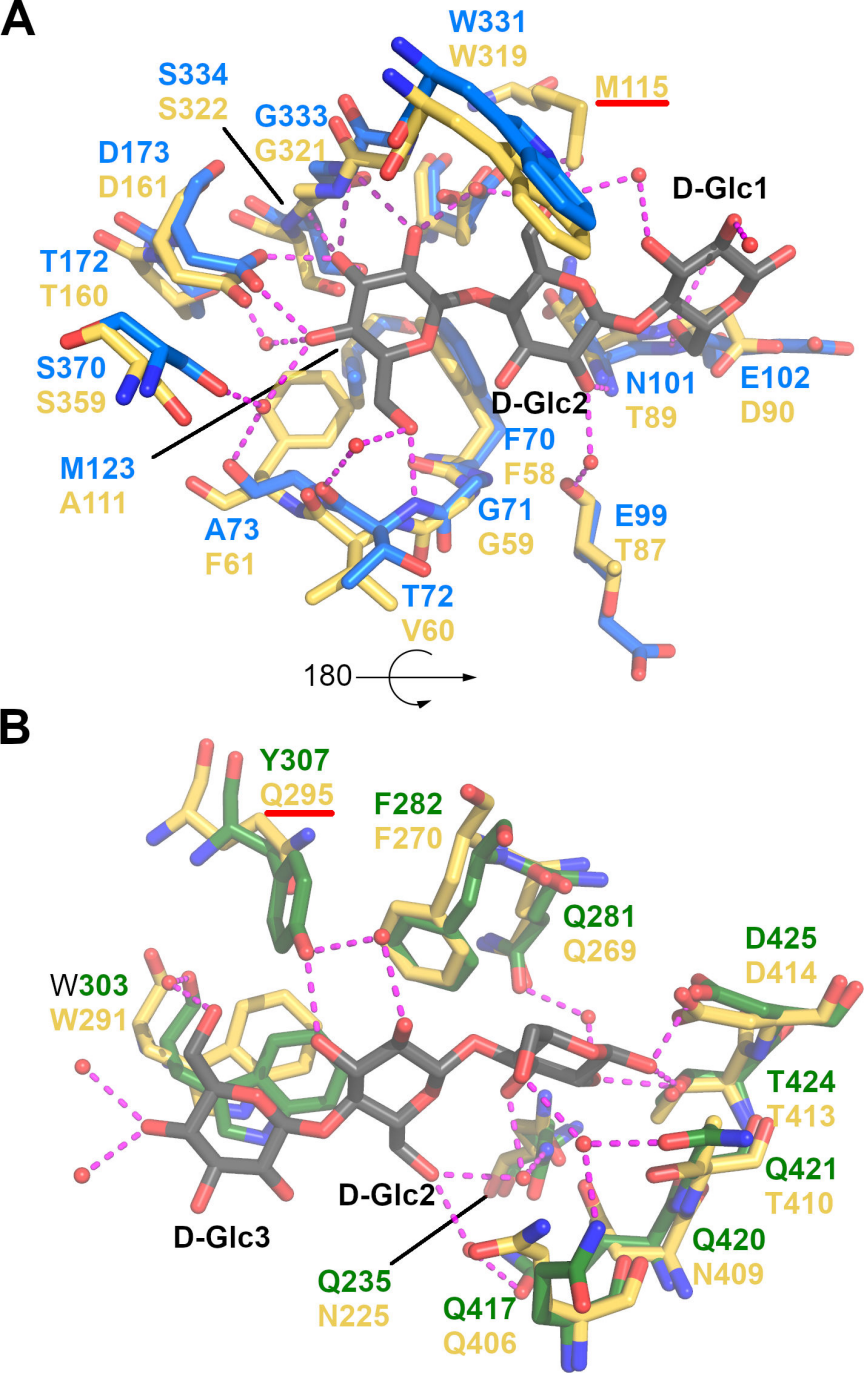
Substrate binding site of the CebE:cellotriose complex superimposed to the CebE^reti^ model. (A) Interactions of residues from Domain 1 (blue sticks) stabilizing cellotriose (gray sticks) superimposed to their equivalent in CebE^reti^ (yellow sticks), water molecules are displayed as small red spheres, and H-bonds as magenta dashed lines. (**B)** Same as panel (**A)** for Domain 2 (green sticks) with a rotation of approximately 180° around a horizontal axis.

**Fig 6:**
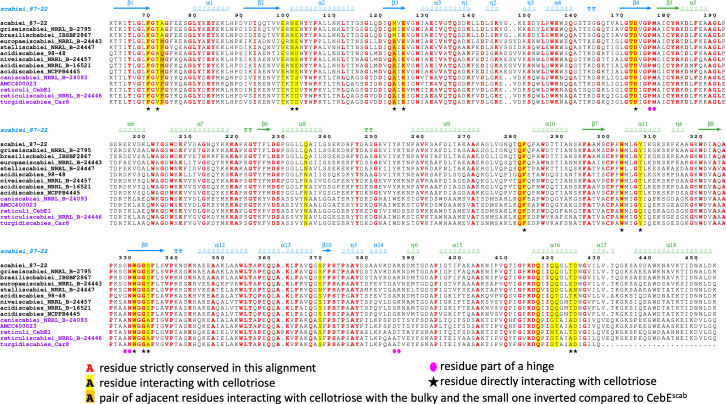
Sequence alignment of CebE proteins from model *Streptomyces* pathogenic species with amino acid numbering of CebE^scab^. Secondary structure elements of the CebEscab:cellotriose structure are schematized above the alignment with the same domain coloring code as in Fig. 1B (Domain 1 in blue and Domain 2 in green), whereas hinge regions are identified with magenta ellipses below the alignment. Residues strictly conserved are shown in red, and those directly interacting with cellotriose or indirectly interacting with cellotriose via a water molecule are highlighted in yellow (or orange when the short and bulky side chains are switched). Stars indicate direct interaction with the ligand. CebE proteins reference IDs: scabiei_87–22 (C9Z451; WP_013003368.1); griseiscabiei_NRRL_B-2795 (MBZ3900963.1); brasiliscabiei_IBSBF2867 (WP_216591689); europaeiscabiei_NRRL_B-24443 (WP_046704818.1); stelliscbiei_NRRL_B-24447 (WP_046918411.1); acidiscabieis_98–48 (WP_075734941.1); niveiscabies_NRRL_B-24457 (WP_055721858.1); acidiscabieis_NRRL_B16521 (WP_029183343.1); acidiscabieis_NCPPB4445 (WP_050369574); caniscabiei_NRRL_B-24093 (WP_060884585.1); AMCC400023 (WP_045557721.1); reticuli_CebE1 (Q9 × 9R7; CAB46342.1); reticuliscabiei_NRRL_B-24446 (WP_059073075.1); and turgidiscabies_Car8 (ELP70267.1).

Ten residues of CebE^scab^ directly or indirectly (via a water molecule) involved in 12 interactions with cellotriose are substituted in CebE^reti^ namely, T72/V60, A73/F61, E99/T87, N101/T89, E102/D90, M123/A111, Q235/N225, Y307/Q295, Q420/N409, and Q421/T410 (Table 2; Fig. 5). Eight of these ten residues are also substituted (by the same or other amino acids) in the CebE proteins of strains that possess a CebE^reti^ background (Fig. 6). Four of these residues, T72, E99, N101, and E102, participate in the H-bond network stabilizing cellotriose through their backbone; their mutation is, therefore, not expected to affect the binding of the ligand. The loss of hydrophobic interaction resulting from the M123/A111 substitution is compensated by the concomitant A73/F61 substitution. The three glutamines (Q235, Q420, and Q421), located close to each other in the structure, interact with cellotriose via H_2_O-mediated H bonds. Their substitution by asparagine (Q235 and Q420) or threonine (Q421) will modify the H-bond network involving water molecules surrounding the ligand. However, the effect on ligand-binding is difficult to evaluate because the number of potential H-bonds is equivalent in CebE^reti^. The two most significant differences between the CebE^scab^ and CebE^reti^ binding site, potentially explaining the reduced affinity for cellotriose, are (i) the Y307/Q295 substitution where the loss of the direct H-bond between Y307 and D-Glc2 is due to the substitution by the shorter glutamine residue and (ii) the G127/M115 substitution that brings a hydrophobic side chain in close proximity with a polar area of the ligand, preventing at least one water mediated H-bond. However, the latter two substitutions are only specific to the CebE protein of *S. reticuli* and are not conserved in the CebE proteins of the CebE^reti^ subgroup found in the pathogenic species *S. turgidiscabies* and *S. caniscabiei* (Fig. 6). Therefore, whether pathogenic species with either a CebE^scab^ or CebE^reti^ background would require different concentrations of elicitor for the onset of thaxtomin production will have to be determined experimentally by characterizing the CebE protein of other *Streptomyces* species from both subgroups as well as by site-directed mutagenesis to address the importance of the different residues contributing to the binding of the ligand. This hypothesis could also imply the highly dynamic ligand binding mechanism of CebE rather than residue substitutions specifically involved in cellotriose binding.

### Conclusions

In this work, we solved the crystal structure of CebE^scab^ in complex with cellotriose at a resolution of 1.55 Å, thereby revealing the structural basis of the first event responsible for root and tuber plant colonization by *S. scabiei*. The interaction between CebE^scab^ and cellotriose involves 26 direct or water-mediated hydrogen bonds and hydrophobic interactions. As previously observed in other sugar-binding proteins of ABC transporters, it is the sugar at the non-reducing end of the oligosaccharide, which occupies the most conserved part of the ligand-binding cleft. An induced-fit mechanism is expected to generate the closed conformational changes of CebE, where cellotriose binding triggers the movement between Domains 1 and 2 of the protein. This mechanism is predicted to facilitate the selection between the unliganded and liganded states of SBPs by the transmembrane domains of the importer (37, 41). Prediction of the CebR regulon revealed that the CebE-mediated import of cellotriose is conserved for triggering the production of thaxtomin phytotoxins in pathogenic *Streptomyces* species. The unique loss of the CebR-repressed expression of thaxtomin biosynthetic genes is found in strains belonging to *S. ipomoeae* species associated with the colonization of sweet potatoes. Based on the sequence similarity between CebE proteins of pathogenic streptomycetes, strains belonging to species *S. acidiscabies*, *S. europeiscabiei*, *S. stelliscabiei*, *S. brasiliscabiei*, *S. griseiscabiei*, and *S. niveiscabiei* would sense the presence of cellotriose with similar affinity as the one previously calculated for CebE^scab^. Instead, pathogenic *Streptomyces* strains of species *S. turgidiscabies* and *S. caniscabiei* possess a CebE protein orthologous to CebE^reti^ with lower affinity for cellotriose, suggesting that they could possibly need a higher quantity of cellotriose released by their host to induce the colonization process. However, this hypothesis would imply the highly dynamic ligand binding mechanism of CebE rather than residues specifically involved in cellotriose binding as the two main substitutions (Y307Q and G127M) possibly responsible for the much lower affinity of CebE^reti^ for cellotriose are not conserved in CebE proteins of strains belonging to species *S. turgidiscabies* and *S. caniscabiei*. Importantly, it has to be noted that our work also provides the structural basis for CebE-mediated uptake of cellobiose and cellotriose by saprophytic non-pathogenic *Streptomyces* species that actively participate in the mineralization of the plant decaying matter.

## Data Availability

The crystallographic structure of CebE in complex with cellotriose has been deposited in the Protein Data Bank with accession number 8BFY.

## SUPPLEMENTAL MATERIAL

Substrate induced closing of the CebE pocket spectrum.01975-23-s0001.docx Table S1, Fig. S1, and legend of Movie S1.

Substrate induced closing of the CebE pocket spectrum.01975-23-s0002.mov Cellotriose induced closing of the CebE pocket.

ASM does not own the copyrights to Supplemental Material that may be linked to, or accessed through, an article. The authors have granted ASM a non-exclusive, world-wide license to publish the Supplemental Material files. Please contact the corresponding author directly for reuse.
