# Supplementary material for: Common scab disease: structural basis of elicitor recognition in pathogenic Streptomyces species
Source: Microbiol Spectr. 2023 Oct 4;11(6):e01975-23. doi: 10.1128/spectrum.01975-23 (PMC10714786; doi:10.1128/spectrum.01975-23)
Supplement: Substrate induced closing of the CebE pocket — Table S1, Fig. S1, and legend of Movie S1. [file spectrum.01975-23-s0001.docx]

**Supplemental material (Kerff et al., 2023)**

**Supplementary Table S1**


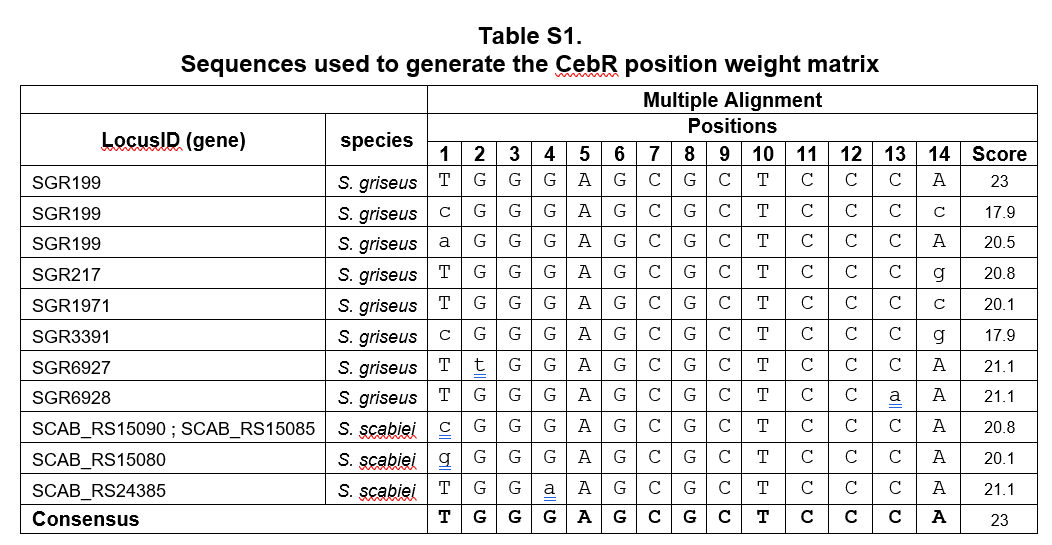


**Supplementary Figure S1**


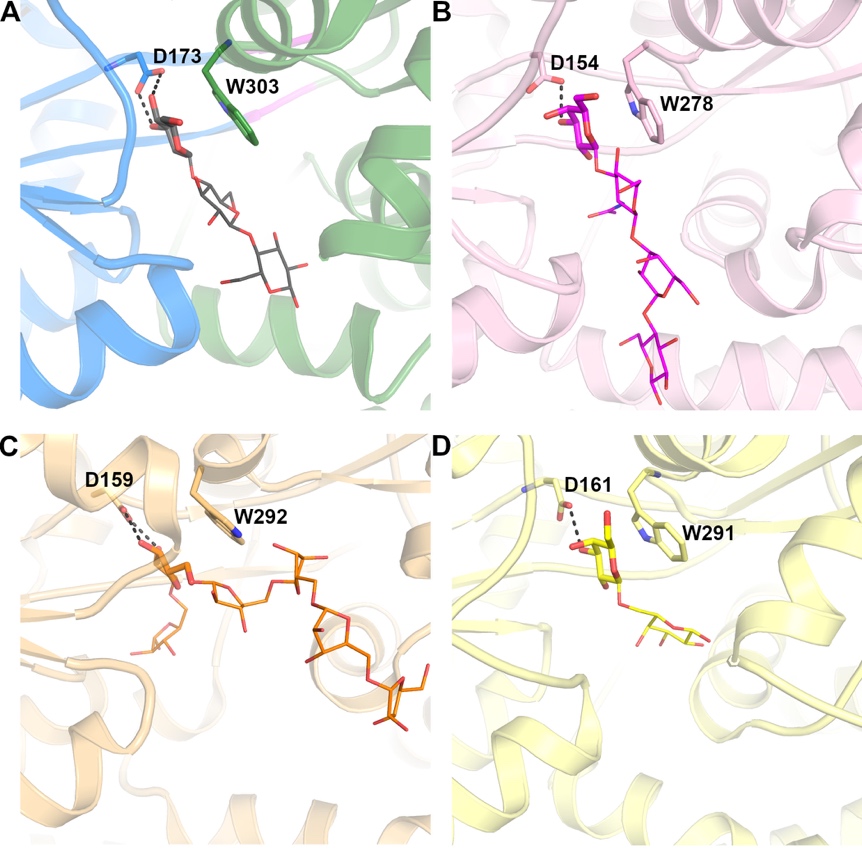


**Figure S1.** **Comparison of saccharide binding mode in four SBP proteins.** **A**. Cartoon representation of the CebE:cellotriose complex with coloring scheme as Figure 2. The aspartate and tryptophan conserved in SBP proteins are represented as sticks. **B**, **C,** and **D**, similar representation for the GL-BP:lacto-N-tetraose (PDB code 2Z8F), AbnE:arabinohexaose (PDB code 6RKH), and Bal6GBP:β-1,6-galactobiose (PDB code 6H0H) complexes, respectively.

**Supplementary movie 1**

Substrate induced closing of the CebE pocket.
